# Predictors of Post-Traumatic Growth in a Sample of United Kingdom Mental and Community Healthcare Workers during the COVID-19 Pandemic

**DOI:** 10.3390/ijerph20043539

**Published:** 2023-02-17

**Authors:** Kirsten Barnicot, Rose McCabe, Angeliki Bogosian, Renos Papadopoulos, Mike Crawford, Peter Aitken, Tanja Christensen, Jonathan Wilson, Bonnie Teague, Ravi Rana, Donna Willis, Ryan Barclay, Amy Chung, Frank Rohricht

**Affiliations:** 1 Department of Health Services Research and Management, University of London, London WC1E 7HU, UK; 2 Department of Psychiatry, Imperial College London, London W12 0NN, UK; 3 Department of Psychosocial and Psychoanalytic Studies, University of Essex, Essex CO4 3SQ, UK; 4 Devon Partnership NHS Trust, Exeter EX2 5AF, UK; 5 Norfolk and Suffolk NHS Foundation Trust, Norwich NR6 5BE, UK; 6 East London NHS Foundation Trust, London E1 8DE, UK

**Keywords:** post-traumatic growth, resilience, occupational health, human resources, COVID-19 pandemic

## Abstract

Experiences of adversity can generate positive psychological effects alongside negative impacts. Little research to date has evaluated predictors of post-traumatic growth in mental or community healthcare workers during the COVID-19 pandemic. Following a survey of 854 community and mental healthcare staff in the United Kingdom in July to September 2020, multiple linear regression was used to determine the association between hypothesised risk and protective factors (personal, organisational and environmental variables) and total scores on the Post-traumatic Growth Inventory–Short Version. Positive self-reflection activities, black and minority ethnic status, developing new healthcare knowledge and skills, connecting with friends and family, feeling supported by senior management, feeling supported by the UK people, and anxiety about the personal and work-related consequences of COVID-19 each significantly independently predicted greater post-traumatic growth. Working in a clinical role and in mental healthcare or community physical healthcare predicted lower post-traumatic growth. Our research supports the value of taking an organisational growth-focused approach to occupational health during times of adversity, by supporting staff to embrace opportunities for personal growth. Valuing staff’s cultural and religious identity and encouraging self-reflective activities, such as mindfulness and meditation, may help to promote post-traumatic growth.

## 1. Introduction

It is widely acknowledged that the coronavirus disease (COVID-19) pandemic has placed healthcare staff under unprecedented stress [1]. Existing research has predominantly focused on the negative effects of the pandemic on staff mental health, with a meta-analysis of sixty-five international studies finding high rates of anxiety (34.4%), depression (31.8%), stress (40.3%), post-traumatic stress (11.4%), insomnia (27.8%), psychological distress (46.1%), and burnout (37.4%) amongst healthcare workers [2].

Stressful life experiences are also known to have the potential to generate positive psychological effects [3]. Healthcare leaders in the United Kingdom (UK) and internationally have stressed the importance of focusing on staff resilience and positive self-development during the COVID-19 pandemic [1,4]. Understanding risk and protective factors for healthcare staff psychological wellbeing has been identified as a COVID-19 research priority [5]. Post-traumatic growth is defined as “positive psychological change experienced as a result of the struggle with highly challenging life circumstances” [3] (p. 1). This phenomenon includes positive changes in personal strength, more meaningful interpersonal relationships, increased appreciation for life, changed priorities, and a richer spiritual life [3]. In a large body of work amongst trauma-exposed individuals, including healthcare workers, military personnel and abuse survivors, post-traumatic growth has been shown to be protective against suicidal ideation, depression, and post-traumatic stress disorder [6,7,8]. Other work suggests a more complex association whereby post-traumatic growth can co-exist with mental health problems [9].

There is a dearth of research on post-traumatic growth in the workplace, and on organisational conditions enabling this [10]. The unprecedented and unexpected challenges for the healthcare workforce brought about by the COVID-19 pandemic provide a unique opportunity to explore factors associated with post-traumatic growth, to facilitate a better understand of its mechanisms and how best to support it. A recent review [11] and our own search identified no studies quantitatively evaluating post-traumatic growth in response to the pandemic amongst healthcare staff in the United Kingdom, and no studies internationally assessing growth specifically in mental health or community healthcare workers. Whilst surveys of healthcare workers in the United States of America and China have identified high levels of post-traumatic growth during the pandemic [12,13], the United Kingdom workforce has faced specific challenges which may have led to a different experience. These include the widely critiqued delayed government response to the pandemic, largescale redeployment of staff within the nationalized public health system, and lower public compliance with measures than in Asian countries, in the context of a workforce already threatened by Brexit and the fourth-lowest number of hospital beds per 1000 population among the G-20 countries [14,15,16,17,18]. Additionally, the pandemic has brought specific challenges for community and mental healthcare staff which merit focus. These staff are not trained in managing infectious disease, yet have been required to care for service users who have COVID-19 and/or are clinically vulnerable, and to prevent the spread of infection among service-users who may struggle to adhere to infection control measures, against a background of a nationwide failure to supply adequate personal protective equipment to healthcare staff [19,20,21,22,23,24,25]. Redeployment of staff, staff illness and the move to remote tele-care saw huge disruptions to mental health and community services [20,22]. At the same time, population mental health worsened, particularly amongst already vulnerable mental health service users [26]. These challenges may be considered a psychological contract breach, whereby health workers’ expectations of what their employer expects of them, and of what obligations their employer has towards them, are transgressed, and a breakdown of trust in one’s employer can result [27,28].

Healthcare organisations, as well as government health policy actors, have a responsibility to protect their staff from burnout and stress and enable them to achieve personal growth in times of adversity [29]. The present research aims to inform organizational policy by identifying personal, organisational and environmental variables acting as risk or protective factors for post-traumatic growth amongst community and mental healthcare staff in the United Kingdom during the COVID-19 pandemic. For instance, environmental risk factors may include exposure to COVID-19 serious illness and deaths, whilst protective environmental factors may include feeling supported by colleagues and the wider community [5]. Our survey aims to overcome the limitations of previous pandemic research on healthcare workers by recruiting through a defined population (National Health Service (NHS) Trusts) rather than social media, and by including both clinical and non-clinical staff [30].

### 1.1. Research Questions and Hypotheses

#### 1.1.1. Question 1. What Is the Association between Post-Traumatic Growth and Sociodemographic and Professional Characteristics?

Hypotheses. No hypotheses were derived regarding sociodemographic characteristics; these were analysed in an exploratory manner. Staff working in inpatient settings may experience lower post-traumatic growth due to being more significantly affected by personal safety considerations and concerns for their patients [23,31]. Conversely, their higher level of pandemic-related stress could promote greater growth through enforcing greater adaptations [32]. Psychologists may experience greater post-traumatic growth due to their increased ability to utilize alternative perspectives on adversity [13,33,34].

#### 1.1.2. Question 2. How Do Exposure to, Risk from, and Anxiety about the COVID-19 Affect Post-Traumatic Growth?

Hypotheses. In a number of studies, greater subjective or objective stress exposure predicts greater post-traumatic growth, through allowing opportunities for changes in mindset, attitude, self-perception and coping behaviours [32]. We hypothesised that greater exposure to, risk from, or anxiety about the COVID-19 pandemic will be linked to greater post-traumatic growth.

#### 1.1.3. Question 3. How Does Connecting with Others Affect Post-Traumatic Growth?

Hypotheses. Evidence suggests that social support promotes post-traumatic growth, particularly when mutual experiences of both stress and growth can be shared [5,35,36,37]. We hypothesise that more time spent connecting with others will be associated with greater post-traumatic growth.

#### 1.1.4. Question 4. How Does Self-Care Affect Post-Traumatic Growth?

Hypotheses. Self-care, i.e., taking care of one’s own holistic wellbeing, is core to the delivery of effective professional mental healthcare, and can prevent burnout [38,39]. Practices which promote self-care include work-life balance, i.e., adequate time away from work, adequate time spent relaxing or doing hobbies [39]. Getting enough sleep and exercise and practicing mindfulness and other forms of self-reflection are also important self-care activities [39]. We hypothesise that staff who take the time to care of themselves will have greater opportunities for personal reflection and for self-development outside of work, promoting greater post-traumatic growth. Further, as found by others, we hypothesise that more positive self-reflection (e.g., mindfulness, meditation, self-help) will be particularly strongly associated with greater post-traumatic growth [40,41,42].

#### 1.1.5. Question 5. How Does Professional Development at Work Affect Post-Traumatic Growth?

Hypotheses. Gaining new knowledge and learning new skills opens up people’s minds to new ways of seeing the world and to a new appreciation of their own abilities [43]. We hypothesise that, as found in a study of frontline nurses in China [44], greater experiences of pandemic-related professional development will be associated with greater post-traumatic growth. 

#### 1.1.6. Question 6. How Does Support at Work Affect Post-Traumatic Growth?

Hypotheses. Occupational support, based in supportive work relationships and enabled by supportive organizational cultures, can play a critical role in helping workers cope with work-related crises and has been shown to promote post-traumatic growth [10,45]. We hypothesise that feeling more practically and morally supported at work will be associated with greater post-traumatic growth. Based on the contention that support from one’s immediate team at work combines the benefit of support from close social contacts, with a shared understanding and learning from the specific challenges faced together at work, we hypothesise that feeling supported by one’s immediate team will be particularly strongly associated with growth. 

#### 1.1.7. Question 7. How Does Psychological Resilience Affect Post-Traumatic Growth?

Hypotheses. In line with previous research, we hypothesise that staff with greater trait psychological resilience will experience greater post-traumatic growth [46,47,48,49,50].

#### 1.1.8. Question 8. How Do Depression and Anxiety Affect Post-Traumatic Growth?

Hypotheses. In line with previous research, we hypothesise that depression and anxiety will be negatively associated with post-traumatic growth [51,52].

## 2. Materials and Methods

### 2.1. Design

Cross-sectional analysis of predictors of post-traumatic growth, using data from the first round of a cohort study.

### 2.2. Ethics Approval and Consent to Participate

The study was approved by the United Kingdom Health Research Authority on 7 July 2020 (ref. 20/HRA/2982). All participants gave written informed consent for participation via a tick-box on the Qualtrics internet-based survey platform.

### 2.3. Setting

The study was conducted across four National Health Service (NHS) Trusts in England: two Trusts specialised in mental health care, whilst two specialised in both mental health and community care. The two mental health care Trusts were in semi-rural localities whilst the two mental health and community care Trusts were based in urban inner London localities. The present analysis uses data collected between 16 July 2020 and 30 September 2020. At the start of the survey, coronavirus cases were falling across the country [53]. Restrictions on pubs, restaurants, leisure facilities and overnight stays had been lifted two weeks prior (4 July 2020). Gatherings of up to thirty people were legally permitted, although the Government was still recommending people avoid gatherings larger than six [54]. Coronavirus rates began rising at the beginning of September 2020 [53]. Two weeks prior to the end of the survey (14 September 2020), restrictions for gathering in England were tightened and people were once again legally prohibited from meeting more than six people socially [54].

### 2.4. Inclusion Criteria

Any member of staff working in any role in one of the participating trusts was eligible to participate. Both clinical and non-clinical staff were included.

### 2.5. Procedure

Research and development personnel in participating NHS trusts sent invitations to participate in the survey to all staff in their trusts, via targeted emails and via inclusion in staff news bulletins, between 16 July 2020 and 30 September 2020. Interested staff were provided with a link to view the participant information sheet, hosted on Qualtrics survey software, and were provided with the research team’s contact details in case they wanted to ask any further questions before deciding whether to participate. Interested staff then completed the consent form and survey online, using Qualtrics. The survey was designed by the authorship team using the measures below.

### 2.6. Measures

- Demographic and professional characteristics, including gender identity, age, ethnicity, British citizenship, healthcare sector, work location, profession, and NHS Trusts, were collected using a standardised pro forma developed by the research team.
- Personal, environmental and organisational risk/protective factors were derived from available academic literature and were collected using a standardised pro forma developed by the research team. These included the following items rated on a binary yes/no scale: self, family or friends becoming seriously ill or hospitalised with COVID-19; frequent workplace exposure to COVID-19; serious illness of local or wider colleagues from COVID-19; self or family classified as clinically extremely vulnerable. A measure of anxiety about the personal and professional effects of COVID-19 was derived from mean scores on the following items, rated on a Likert scale from Not at all (1), A little bit (2), Somewhat (3) to A lot (4): Worry about yourself or loved ones becoming seriously ill or dying from COVID-19; Worry about colleagues becoming seriously ill or dying from COVID-19; Worry about service-users becoming seriously ill or dying from COVID-19; Worry about your service-users becoming seriously ill or dying from other serious mental or physical health conditions; Feeling less able to deliver adequate and safe mental health care; Feeling less able to deliver adequate and safe physical health care (α = 0.78). These items were based on contemporary academic and professional body publications outlining the specific concerns of healthcare and mental healthcare workers [19,20,21,22,23]. At the time, no validated scale to assess these concerns existed. The following items related to potential protective factors, measured on the same 4-point Likert scale, were also assessed as individual predictors: Time connecting with friends and family; Time connecting with colleagues; Time relaxing or doing hobbies; Time away from work; Time away from thinking about work; Time spent exercising; Time spent on positive self-reflection e.g., mindfulness, meditation, self-help; Adequate and restful sleep; Developed new knowledge or skills in delivering mental health care; Developed new knowledge or skills in delivering physical health care; Access to adequate infection control measures; Access to adequate personal protective equipment; Felt supported by my immediate team; Felt supported by senior management; Felt supported by the United Kingdom government; Felt supported by the people of the UK.
- Psychological resilience was measured using the Connor–Davidson Resilience Scale—10 item Version [55], (α = 0.88).
- Depression was measured using the Patient Health Questionnaire—9 item version (PHQ-9) [56], (α = 0.88).
- Anxiety was measured using the Generalised Anxiety Disorder Assessment—7 item version (GAD-7) [57], (α = 0.92).
- Post-traumatic growth was measured using the 10 item Post-traumatic Growth Inventory—Short Version [58], (α = 0.91).
- Adversity-activated development was assessed qualitatively using the Adversity Reflection Tool [59,60,61], the findings from which are beyond the scope of the present analysis.

### 2.7. Analysis

Linear regression in STATA version 14.2 was used to determine the association between individual predictors and total scores on the Post-traumatic Growth Inventory—Short Version. To adjust for multiple testing, the Holm–Bonferroni correction was calculated in Excel to produce adjusted *p*-values [62]. Subsequently, multiple linear regression modelling with forward selection was used to evaluate whether inclusion of additional predictors improved explanatory power and goodness of fit. Only variables significantly associated with the dependent variable in single regression models were added, in order of their R^2^ explanatory power in the single regression models. Variables that were highly conceptually inter-related and had similar R^2^ values in single regression models (±0.02) were entered simultaneously. In a final step, variables that were non-significant in the full multiple regression model were removed and the Akaike and Bayesian information criteria used to establish whether this improved the fit of the model to the data.

## 3. Results

### 3.1. Response Rates

A total of 854 staff took part, from a total potential sample of 24,800 staff members across participating NHS Trusts (data provided by Trust Research and Development departments). Thus, the survey response rate was 3%.

### 3.2. Participant Characteristics

The mean age of participants was 46.68 years (11.24). The majority were female (81.7%) and White British (73.4%). The majority of the participants worked in mental health (88.2%), with 7.8% working in community physical health and 4% working in primary care. Half of the sample worked in a community setting (e.g., clinics, patient homes) (51.1%), 13.6% worked in inpatient services, 10.9% worked both in community and inpatient, and 24.4% worked at administration offices. Participants’ professions varied and included administration (17.2%), consultant/SAS medical doctor (3.9%), allied health professional (12.8%), psychology/psychotherapy (18%), healthcare assistant/support worker (5.6%), nursing/midwifery (24.8%), social worker (3.2%), management (6.4%), other (5.3%), trainee doctor (0.7%), scientific and technical professions (0.5%), pharmacy (0.6%), and additional clinical services (1.1%). Participant mean age and gender distribution was similar across Trusts. The two urban London trusts had a substantially higher proportion of ethnic minority participants (43% and 51% respectively) compared to the semi-rural Trusts (9% and 14% respectively). The average total and item scores on the brief post-traumatic growth inventory were 20.35 (s.d. 10.92) and 2.03 (s.d. 1.09) respectively. Twenty-one percent of staff reported moderate to high levels of growth (PTGI item mean score ≥3) [63].

### 3.3. Individual Predictors of Post-Traumatic Growth

Findings from the linear regression models of individual predictors of post-traumatic growth are shown in Table 1, grouped by research question, and are summarised below.

#### 3.3.1. Question 1. What Is the Association between Post-Traumatic Growth and Sociodemographic and Professional Characteristics?

Higher post-traumatic growth was significantly associated with Asian or Black ethnicity, working in a non-clinical role and/or in administrative offices and/or in primary care, and working for a particular NHS Trust (located in London). Contrary to the hypothesis, working in an inpatient setting was not linked to lower post-traumatic growth, whereas working in a community setting was. Contrary to the hypothesis, allied health professionals, nursing staff and psychology staff reported less post-traumatic growth than non-clinical staff. The variable with the highest explanatory power was ethnicity, explaining 9% of the variance in post-traumatic growth. Age and British citizenship were not related to post-traumatic growth.

#### 3.3.2. Question 2. How do Exposure to, Risk from, and Anxiety about the COVID-19 Pandemic Affect Post-Traumatic Growth?

The average score on our COVID-19 anxiety measure was 2.60 (s.d. 0.57), suggesting that on average participants were “somewhat worried” about the personal and work-related consequences of the COVID-19 pandemic. As hypothesised, greater personal/familial exposure to or risk from COVID-19 and increased anxiety about COVID-19 were each linked to greater post-traumatic growth. Workplace exposure to COVID-19 was not related to post-traumatic growth.

#### 3.3.3. Question 3. How Does Connecting with Others Affect Post-Traumatic Growth?

As per the hypothesis, more time spent connecting with others was associated with greater post-traumatic growth.

#### 3.3.4. Question 4. How Does Self-Care Affect Post-Traumatic Growth?

As per the hypothesis, more time spent on self-care (relaxing, exercising, positive self-reflection) was generally associated with greater post-traumatic growth, with time spent on positive self-reflection a particularly strong predictor. Time spent away from work, time not thinking about work, and adequate sleep were not significantly related to growth. 

#### 3.3.5. Question 5. How Does Professional Development at Work Affect Post-Traumatic Growth?

As per the hypothesis, greater professional development at work was linked to greater post-traumatic growth.

#### 3.3.6. Question 6. How Does Support at Work Affect Post-Traumatic Growth?

As hypothesised, feeling supported at work was consistently associated with greater post-traumatic growth. Whilst feeling supported by one’s immediate team was linked to greater growth, this was not the strongest predictor, with feeling support from senior management, the UK government and the people of the UK being most highly predictive. Contrary to hypothesis, practical support at work in the form of infection control and access to PPE was not linked with growth.

#### 3.3.7. Question 7. How Does Psychological Resilience Affect Post-Traumatic Growth?

Contrary to the hypothesis, psychological resilience was not associated with post-traumatic growth.

#### 3.3.8. Question 8. How Do Depression and Anxiety Affect Post-Traumatic Growth?

Contrary to the hypothesis, depression and anxiety were not associated with post-traumatic growth.

### 3.4. Combination of Predictors Best Explaining Post-Traumatic Growth

Time spent on positive self-reflection and ethnicity jointly explained the most variance in single regression models (both R^2^ = 0.09). Time spent on positive self-reflection was added first due to its strong conceptual relationship with post-traumatic growth (Model 1). The addition of ethnicity led to a significant increase in the explanatory power of the model, almost doubling the R^2^ to 0.17 (Model 2), indicating that ethnicity has a substantial association with post-traumatic growth that is largely independent of the effect of positive self-reflection. In subsequent models (Models 3, 4, 6, 7, and 8), the addition of variables related to professional skills development, connecting with others, feeling supported at work, professional characteristics, and exposure to or risk of COVID-19 each led to a significant increase in the explanatory power of the model, with the full model holding a total adjusted R^2^ of 0.31. An exception was the addition of variables related to other self-care (time spent relaxing and doing hobbies, exercise), which did not add significant additional explanatory power to the model (Model 5). In a final model (Model 9), variables that were non-significant in earlier multiple regression models were dropped. This did not lead to any drop in explanatory power, explaining 31% of the variance in post-traumatic growth, and yielded superior Akaike and Bayesian information criteria over the full model, indicating it offered the best balance of explanatory power versus parsimony and hence provided the best fit to the data. The final model is shown in Table 2 below, whilst findings from Models 1–8 are displayed in Online Supplementary Information Table S1. In the final model, time spent on positive self-reflection, Asian or black ethnicity, developing new knowledge and skills in physical healthcare or mental healthcare, connecting with friends and family, feeling supported by senior management, feeling supported by the people of the UK, and anxiety about the personal and work-related consequences of COVID-19 each significantly independently predicted greater post-traumatic growth. Working in a clinical role, and in mental healthcare or community physical healthcare, predicted lower post-traumatic growth, with the largest negative effects seen amongst psychology and psychotherapy staff.

## 4. Discussion

### 4.1. Summary of Main Findings

In a survey of 854 staff across four United Kingdom mental health and community care NHS Trusts, mean post-traumatic growth scores were lower than in a sample of frontline COVID-19 nurses in Wuhan, China [44], but equivalent to a sample of Iraqi war veterans [64]. Time spent on positive self-reflection activities and black and minority ethnic status each had a large and independent positive effect on post-traumatic growth. Developing new knowledge and skills in physical healthcare or mental healthcare, connecting with friends and family, feeling supported by senior management, feeling supported by the people of the UK, and anxiety about the personal and work-related consequences of COVID-19 each significantly independently predicted greater post-traumatic growth. Working in a clinical role, and in mental healthcare or community physical healthcare, predicted lower post-traumatic growth.

To our knowledge, this is the first study quantitatively evaluating post-traumatic growth in response to the pandemic amongst healthcare staff in the United Kingdom, and the first worldwide in mental health and community healthcare workers. Whilst previous international studies have examined predictors of pandemic-related post-traumatic growth, they have addressed a more limited range of predictors and hence have built a less complete picture [11]. Our analysis suggests potential implications for what healthcare organisations can do to promote worker growth and continuous development in the post-pandemic phase. This focus on growth and development adds to previous recommendations for supporting healthcare staff post-pandemic, which have instead focused primarily on organizational support for staff experiencing mental health problems, improving health and safety and working conditions, or on supporting staff to adapt to new technologies and hybrid ways of working [65,66,67].

### 4.2. Interpretation of Findings and Implications for Organisational Management

#### 4.2.1. Positive Self-Reflection

Our findings on the value of positive self-reflection are congruent with a number of studies showing that engaging in mindfulness and meditative practice can enhance post-traumatic growth [40,68,69]. Spending time mentally stepping back from one’s experiences and observing one’s mental processes can allow space for consideration of multiple viewpoints on adversity, including a search for meaning, reflection on positive outcomes, and a determination to thrive [40,70]. The findings suggest that employee growth can be promoted by encouraging staff to undertake positive self-reflection activities, such as mindfulness and meditation, and providing access to regular guided teaching and practice. Organisations should facilitate staff to reflect on how they have been affected by the pandemic, encourage viewing the pandemic as both a crisis and as an opportunity to improve on the status quo, and provide opportunities for reflective processing of traumatic memories and anxieties in a supportive context [12,71]. Specific interventions that are helpful for facilitating this type of reflective processing and a growth mindset may include the use of appreciative enquiry during supervision, which involves asking staff to share narratives about times when the organization and its people were successful and satisfied, together with questions about the future to help envision strategies and action towards positive change [72]. Relatedly, solution-focussed problem solving in team supervision may facilitate staff to focus on their strengths and the small steps they can take to enact positive change [73].

#### 4.2.2. Ethnicity

Corresponding with our findings, a number of studies conducted in the USA have found that non-white ethnicity predicts greater post-traumatic growth, including in flood survivors, cancers survivors, sexual assault survivors, military veterans, and older adults and frontline healthcare workers during the COVID-19 pandemic [12,74,75,76,77,78]. Black and minority ethnic healthcare staff in our study were exposed to a wealth of evidence via the media that they were at higher risk of serious illness or death if they contracted COVID-19. Additionally, the majority of participating black and minority ethnic staff worked for our two inner city NHS Trusts, both of which set up black and minority ethnic staff support networks and facilitated team discussions around racism and racial diversity during the pandemic. These initiatives occurred in part in response to the growing public awareness and support for the Black Lives Matter movement following the death of George Floyd. Given the findings of ourselves and others that higher stress or exposure to adversity, and feeling more supported at work, predict greater post-traumatic growth, these factors may offer a partial explanation for our findings. However, the effect of ethnicity on post-traumatic growth was independent and larger than that of exposure/anxiety about COVID-19 or feeling supported at work, suggesting that these factors cannot fully explain the positive effects of ethnicity.

A further explanation could be the likelihood of greater religiosity and community identity amongst black and minority ethnic staff. In the UK, black and minority ethnic people are far more likely than their white counterparts to actively practice religion (e.g., regular attendance at places of worship), and to see religion as a strong marker of their identity [79]. Religious identity can bring meaning and resilience to both the practice of healthcare and coping with adversity [80,81,82]. Previous research has found that greater religiosity and spirituality predicts greater post-traumatic growth [78,83,84], and mediates the positive effect of ethnicity on growth [75]. Holding a strong religious and/or cultural identity can also promote a sense of belonging and social connectedness within one’s own community [85]. Further, it has been suggested that previous experiences of coping with racism together as a community may prime black and minority ethnic communities to cope positively with adversity [86,87]. Our findings suggest that organisations can promote personal growth by valuing and encouraging staff’s sense of community and identity, including cultural and religious identity, in coping with adversity. Specific actions could include creating staff networks for people with minority cultural or religious identities. In the UK NHS, such networks have been shown to help promote a sense of solidarity and create a ‘psychological safe space’ for discussing inclusion, diversity and equality issues [88]. Further, organizational engagement with initiatives, such as Black History Month [89] and Black Lives Matter [90], to celebrate the societal contributions and achievements of under-represented groups, may facilitate minority staff to consider the role of their ethnic and community identity as a source of pride and personal growth. Further, in providing structured and supportive opportunities for staff to reflect on how they have been affected by the pandemic, organisations should explicitly encourage staff to reflect on how their racial and cultural identities have both exposed them to greater risks and adversities [91] whilst also providing a source of strength and community towards personal growth [79,80,81,82,83,84,85,86,87].

#### 4.2.3. Professional Development

As much as the pandemic brought challenges for safe and effective healthcare, it also necessitated healthcare staff learning new skills, including the use of video technologies for delivering remote assessment and training, enhanced infection control competencies, and, in some cases, redeployment into a new area [20,22,92]. Where employees felt ill-equipped to deal with these changes, this could contribute to a sense of organizational injustice and a perceived breach of the psychological contract with their employers, leading to reduced post-traumatic growth [27,93]. Relatedly, healthcare organisations with low levels of pandemic resilience were characterised by a lack of support for their workers to adapt and learn new skills [94]. Conversely, where employees were equipped by their employees to use these new experiences to gain new knowledge and learn new skills, we and others have found that such experiences provide an opportunity for positive personal growth [44,95]. This complements previous research showing that, post-pandemic, the wellbeing of workers providing hybrid healthcare is optimized by leadership that promotes employee adaptability and personal growth [96]. Similarly, guidance for promoting the mental health of healthcare workers post-pandemic has emphasized that a commitment to continuing professional development, including maintaining at least a basic programme during extended periods of pressure, gives staff a sense of hope and progression and can promote mental health resilience [65]. Additionally, research has shown that training employees in new skills may be more likely to result in personal growth where trainers and organizations cultivate a “growth mindset”, i.e., a belief in the capacity of individuals and organizations to learn from mistakes and find innovative solutions in new ways of working [97,98].

#### 4.2.4. Social Support

Connecting with friends and family and feeling supported by senior management and the people of the UK each independently predicted greater post-traumatic growth. According to Calhoun and Tedeschi [99], cognitive processing of traumatic events can be fostered by sharing one’s internal experience with others who are able to offer support. Evidence suggests that social support promotes post-traumatic growth, particularly when mutual experiences of both stress and growth can be shared [35,36,37]. A more basic explanation is that, for some healthcare workers working from home, the pandemic afforded growth due to positive experiences of being able to spend more time with loved ones [100].

Contrary to the hypothesis, but congruent with research on Dutch peacekeepers [74], feeling supported by one’s immediate team did not explain additional variance above the effect of support from senior management and the UK people. This reinforces the important role of management and organisational culture in promoting growth [10], congruent with evidence that perceived organisational support prevented post-traumatic stress disorder (PTSD) in frontline healthcare workers during the pandemic [101]. By contrast, staff burnout and emotional exhaustion has been linked to decreased post-traumatic growth [49]. An organizational climate that supports continuous professional development and new innovations to promote staff wellbeing is key to both individual job satisfaction and organizational sustainability [102]. Such a climate can be created by a transformational leadership style, which focusses on inspiring and motivating employees to think creatively and to feel empowered to enact positive change in their organization [103], and has been shown to reduce burnout and increase job satisfaction and innovative ways of working [104,105,106]. This is in turn facilitated by a clear leadership vision, leadership behaviour which builds trust and respect, and communication with staff that encourages knowledge sharing, collaboration, and creativity [107]. Similarly, guidance on promoting healthcare staff wellbeing post-pandemic emphasizes creating a culture of effective and honest communication between managers and staff, where staff are encouraged to express their ideas and innovations locally [65].

Further, our survey took place when public attitudes to pandemic healthcare workers were resoundingly positive, with the weekly public “Clap for carers” initiative having recently ended, and NHS rainbow artwork still festooning many residential and commercial windows [108]. Whilst some evidence suggests that the public elevation of healthcare workers to hero status was mostly viewed with skepticism by professionals [109,110], and tarred by the escalation of verbal and physical attacks later in the pandemic [111], our findings suggest that the perception of public support as genuine promoted positive growth. Public support was mainly aimed at hospital staff and emergency workers. Our analysis suggests that mental health and community professionals were also able to benefit from this.

#### 4.2.5. Anxiety about the Personal and Work-Related Consequences of COVID-19

Staff scores on our COVID-19 anxiety measure represent a moderate level of anxiety about themselves, loved ones, colleagues, and their service-users becoming seriously ill or dying from COVID-19, as well as worry about service-users becoming more unwell for other reasons whilst staff feel less able to deliver an adequate level of care. In the UK and elsewhere, mental health and community workers were tasked with preventing the spread of infection amongst patients and staff, with inadequate training and less access to PPE provision than other staff groups [19,20,21,22,23,24]. They were also tasked with monitoring and risk managing the mental health of service-users remotely, in the context of many service users experiencing an exacerbation of their mental health difficulties due to the pandemic. These concerns were also reported in other surveys of mental health staff to be a major cause of stress and anxiety [25], and to potentially represent a breach of the psychological contract with their employers and the UK government [27]. However, rather than this being a wholly negative experience, our findings are congruent with a large body of work suggesting that greater subjective or objective stress exposure predicts greater post-traumatic growth [32]. Similarly, fear of contagion predicted greater post-traumatic growth amongst Spanish healthcare workers during the pandemic [112]. Some research suggests a U-shaped relationship whereby medium exposure to stress predicts greater growth than low or high exposure [32]. We may conjecture that the maximum level of stress experienced in our sample was moderate compared to higher-stress trauma populations where an adverse effect on growth has been demonstrated, such as Dresden bombing victims and bereaved parents [113,114]. This finding ties in with other work suggesting that experiencing stress and the reflective processing of these stressful experiences may be a necessary precursor of post-traumatic growth [115,116,117]. This reinforces our earlier suggestion that organisations should encourage viewing the pandemic as both a crisis and as an opportunity to improve on the status quo and provide opportunities for reflective processing of traumatic memories and anxieties in a supportive context [12,71].

#### 4.2.6. Psychological Resilience and Mental Health

Contrary to the hypothesis and some international studies of healthcare workers during the pandemic [47,49], psychological resilience was not related to post-traumatic growth. Our findings are potentially congruent with other research showing a more complex relationship, whereby resilience only promotes positive growth if the stressor is perceived as a challenge and not as a threat [118]. Others have argued that individuals with high levels of pre-existing resilience may have less capacity for post-traumatic growth than less resilient individuals, since they experience less distress in the face of adversity [119]. Our findings are also commensurate with previous research showing that growth can co-exist independently of anxiety and depressive mood states [9]. However, other research has painted a more complex picture whereby anxiety can in some cases promote growth whilst depression can inhibit it, and growth may act as a protective factor against future mental distress [9].

#### 4.2.7. Strengths and Limitations

This was, to our knowledge, the first published evaluation of post-traumatic growth amongst UK healthcare personnel during the COVID-19 pandemic, and the first international evaluation of post-traumatic growth specifically amongst mental health and community staff. Strengths include sampling via NHS trusts rather than through social media, arguably resulting in less selection bias [30], and the inclusion of both urban and semi-rural localities, and staff directly involved in care and those not. These factors are likely to have increased the representativeness of our findings. Similarly, the proportion of black and minority ethnic staff in the sample (27%) is representative of that within the wider UK NHS workforce (22%) [120] and within the participating trusts, as is our proportion of male staff participants (23% in the wider UK NHS workforce) [121].

Limitations include our low response rate (3%). Healthcare workers busy dealing with the pandemic and all of its associated stresses may have missed the recruitment emails or may have been put off by the advertised survey completion time (15 to 20 min including a reflection activity not described here). Thus, potentially, the staff who did respond may be a sample that were particularly motivated to reflect on positive and negative pandemic effects. However, low response rates have been found to exert only minimal effects on statistical associations between variables in surveys [122], which is the focus of our analysis. Additionally, there is a possibility of recall bias and confounding, whereby people were asked to comment simultaneously on their growth and factors such as managerial support, making it difficult to establish the direction of causality. Further, there could be other unmeasured factors such as temperament that explain some of the identified associations.

#### 4.2.8. Implications for Further Research

Further research should explore why black and minority ethnic identity may help to promote growth, including an investigation of how a sense of community belonging and religious identity may be beneficial, but also drawing on qualitative data to better understand the complexity of black and minority healthcare staff’s experiences of the pandemic. Similarly, drawing on healthcare staff’s qualitative accounts of their experiences of the pandemic could better elucidate what specific management behaviours were beneficial in promoting growth. Finally, further potential predictors of post-traumatic growth, such as religiosity, coping skills and post-traumatic stress, should be tested in order to explain additional variance. Future research should assess the course of post-traumatic growth over the pandemic as the findings identified here reflect post-traumatic growth at the peak of the pandemic, where healthcare staff were experiencing both the highest levels of stress and equally the greatest prospects for growth through adaptation to new circumstances. Evaluating prospective predictors and consequences of post-traumatic growth would better elucidate the directionality of associations and enable a better understanding of whether post-traumatic growth exerts a protective influence on future mental health.

## 5. Conclusions

A resilient organization “builds and uses its capability endowments to interact with the environment in a way that positively adjusts and maintains functioning prior to, during, and following adversity” [123] (p. 742). Our work supports the value of thinking beyond organisational resilience to consideration of organisational post-traumatic growth [88]. Our research supports the value of taking an organizational growth-focused approach to occupational health during times of adversity, such as the COVID-19 pandemic, by supporting staff to build on their strengths and identify and engage with opportunities for personal growth and professional development, rather than solely focussing on support for staff struggling with their mental health [124,125]. In particular, valuing the role of staff’s cultural and religious identity in their work and in coping with adversity, and supporting staff to engage in reflective processing of stressful events through activities, such as mindfulness and meditation, may help to promote post-traumatic growth.

## Figures and Tables

**Table 1:** Individual predictors of post-traumatic growth (total score on the brief Post-Traumatic Growth Inventory).

	B	95% CI	Unadjusted *p*	Adjusted *p*	R^2^
Age	0.04	−0.02 to 0.11	0.19	1.00	<0.001
Male gender identity	−2.19	−4.2 to −0.18	0.03	0.51	0.01
Ethnicity					0.09
Vs. White:					
Asian	10.8	7.6 to 14.1	<0.0001	<0.0001	
Black	12.3	8.4 to 16.3	<0.0001	<0.0001	
Mixed	2.27	−1.22 to 5.74	0.20	1.00	
British Citizenship					0.01
Not a British citizen	2.34	−3.95 to 5.07	0.09	1.00	
Healthcare sector					0.01
Vs. mental health:					
Community physical health	1.29	−1.49 to 4.08	0.36	1.00	
Primary care	5.76	2.06 to 9.46	<0.001	<0.0001	
Location of work					0.02
Vs. Administration offices					
Community only	−3.59	−5.25 to −1.74	<0.0001	<0.0001	
Inpatient (or mixed)	−1.60	−3.76 to 0.56	0.15	1.00	
Profession					0.04
Vs. non-clinical					
Any clinical	−4.35	−6.12 to −2.60	<0.0001	<0.0001	
AHPs & social workers	−4.49	−6.94 to −2.05	<0.0001	<0.0001	
HCAs & support workers	−2.48	−6.21 to 1.25	0.19	1.00	
Medical	−1.49	−5.05 to 2.07	0.41	1.00	
Nursing & midwifery	−4.04	−6.12 to −1.96	<0.0001	<0.0001	
Psychology & psychotherapy	−5.98	−8.19 to −3.77	<0.0001	<0.0001	
NHS Trust					0.02
Vs. Trust 1					
Trust 2	−3.04	−4.96 to −1.12	0.002	<0.0001	
Trust 3	−3.47	−5.55 to −1.38	0.001	<0.001	
Trust 4	−2.53	−4.86 to −1.20	0.033	0.51	
Self, family or friends becoming seriously ill or hospitalised with COVID-19	3.39	1.36 to 5.41	<0.0001	<0.0001	0.01
Frequent workplace exposure to COVID-19 or serious illness of local or wider colleagues from COVID-19	1.22	−2.4 to 2.69	0.10	1.00	0.01
Self or family CEV	3.61	1.20 to 6.01	<0.0001	<0.0001	0.01
Extent of anxiety about the personal and work-related consequences of COVID-19	3.29	1.96 to 4.63	<0.0001	<0.0001	0.03
Time spent connecting with colleagues	2.74	1.89 to 3.58	<0.0001	<0.0001	0.05
Time spent connecting with friends and family	2.98	2.00 to 3.77	<0.0001	<0.0001	0.05
Time spent relaxing or doing hobbies	1.97	1.13 to 2.80	<0.0001	<0.0001	0.03
Time spent away from work	0.96	0.05 to 1.87	0.04	0.64	0.01
Time spent not thinking about work	0.01	−0.85 to 0.87	0.99	1.00	0.00
Time spent exercising	2.06	1.24 to 2.87	<0.0001	<0.0001	0.03
Time spent on positive self-reflection	3.61	2.86 to 4.37	<0.0001	<0.0001	0.09
Adequate and restful sleep	0.57	−0.29 to 1.43	0.19	1.00	0.00
Developed new knowledge or skills in delivering mental health care	2.53	1.80 to 3.26	<0.0001	<0.0001	0.05
Developed new knowledge or skills in delivering physical health care	3.70	2.90 to 4.49	<0.0001	<0.0001	0.09
Support at work					
Had access to adequate infection control measures	0.27	−0.51 to 1.04	0.50	1.00	0.00
Had access to adequate personal protective equipment	0.00	−0.73 to 0.73	0.99	1.00	0.00
Felt supported by my immediate team	1.72	0.83 to 2.62	<0.0001	<0.0001	0.02
Felt supported by senior management	1.76	1.02 to 2.52	<0.0001	<0.0001	0.03
Felt supported by the UK government	2.23	1.37 to 3.08	<0.0001	<0.0001	0.03
Felt supported by the people of the UK	2.13	1.26 to 3.00	<0.0001	<0.0001	0.03
Psychological resilience (CDRS-10)	0.23	−0.79 to 1.25	0.66	1.00	0.00
Depression (PHQ-9)	−0.87	−0.24 to 0.06	0.25	1.00	0.00
Anxiety (GAD-7)	0.09	−0.07 to 0.24	0.27	1.00	0.00

AHP Allied Health Professional; CDRS-10 Connor-Davidson Resilience Scale—10 item Version; CEV Clinically extremely vulnerable; GAD-7 Generalised Anxiety Disorder Assessment—7 item version; HCA Healthcare Assistant; NHS National Health Service; PHQ-9 Patient Health Questionnaire—9 item version; UK United Kingdom.

**Table 2:** Final multiple regression model (Model 9).

Model: Total Adjusted R^2^ = 0.32	B	95% CI	*p*
Time spent on positiveself-reflection	2.17	1.40 to 2.94	<0.0001
Ethnicity			
Vs. White:			
Asian	7.03	3.78 to 10.27	<0.0001
Black	11.21	7.82 to 14.60	<0.0001
Mixed	2.85	−0.65 to 6.34	0.11
Developed new skills in physical healthcare	2.13	1.23 to 3.04	<0.0001
Developed new skills in mental healthcare	1.05	0.22 to 1.88	0.013
Time spent connecting with friends and family	1.89	1.10 to 2.67	<0.0001
Felt supported by senior management	0.89	0.20 to 1.58	0.012
Felt supported by the people of the UK	1.46	0.70 to 2.21	<0.0001
Profession			
Vs. non-clinical			
AHPs & social workers	−5.60	−7.77 to −3.42	<0.0001
HCAs & support workers	−3.56	−6.69 to −0.44	0.026
Medical	−4.92	−8.40 to −1.44	0.006
Nursing & midwifery	−5.24	−7.22 to −3.26	0.0001
Psychology & psychotherapy	−6.52	−8.67 to −4.83	<0.0001
Healthcare sector			
Vs. primary care:			
Community physical health	−4.16	−8.21 to −0.11	0.044
Mental health	−3.68	−7.06 to −0.31	0.032
Extent of anxiety about the personal and work-related consequences of COVID-19	2.10	0.88 to 3.32	0.001

## Data Availability

The datasets generated and/or analysed during the current study are not publicly available due to confidentiality and consent concerns, but are available from the corresponding author on reasonable request.

## Supplementary Materials

The following supporting information can be downloaded at: https://www.mdpi.com/article/10.3390/ijerph20043539/s1, Table S1: Multiple regression models 1 to 9.
