# Supplementary material for: Predictors of Post-Traumatic Growth in a Sample of United Kingdom Mental and Community Healthcare Workers during the COVID-19 Pandemic
Source: Int J Environ Res Public Health. 2023 Feb 17;20(4):3539. doi: 10.3390/ijerph20043539 (PMC9965513; doi:10.3390/ijerph20043539)
Supplement: Supplementary file 1 [file ijerph-20-03539-s001.zip › ijerph-2202981-supplementary.pdf]

Supplementary Online Information Table S1. Multiple regression models 1 to 9

		Adjusted R-squared	Likelihood ratio test
Model 0<br>Empty model	$R^2 = 0.00$<br>B = 20.35, 95% CI 19.6 to 21.1, p < 0.0001	0.00	
Model 1 Self-reflection<br>Time spent on positive self-reflection	$R^2 = 0.09$<br>B = 3.61, 95% CI 2.86 to 4.37, p < 0.0001	0.09	X^2 = 84.3, p < 0.0001
Model 2 Ethnicity<br>Time spent on positive self-reflection<br>Ethnicity<br>Vs. White:<br>Asian<br><br>Black<br><br>Mixed	$R^2 = 0.17$<br>B = 3.28, 95% CI 2.55 to 4.02, p < 0.0001<br><br><br>B = 9.76, 95% CI 6.68 to 12.82, p < 0.0001<br>B = 11.36, 95% CI 7.97 to 14.75, p < 0.0001<br>B = 2.24, 95% CI -1.40 to 5.88, p = 0.23	0.17	X^2 = 75.09, p < 0.0001
Model 3 Skills development<br>Time spent on positive self-reflection<br><br>Ethnicity<br>Vs. White:<br>Asian<br><br>Black<br><br>Mixed<br><br>Developed new skills in physical healthcare<br><br>Developed new skills in mental healthcare	$R^2 = 0.22$<br><br>B = 2.62, 95% CI 1.86 to 3.38,p < 0.0001<br><br><br><br>B = 8.58, 95% CI 5.54 to 11.61, p < 0.001<br>B = 10.54, 95% CI 7.20 to 13.89, p < 0.0001<br>B = 2.09, 95% CI -1.45 to 5.62, p = 0.25<br>B =2.56, 95% CI 1.73 to 3.44, p < 0.0001<br><br>B = 0.81, 95% CI 0.04 to 1.58, p = 0.04	0.22	X^2 = 59.31, p < 0.0001
Model 4 Connecting with others<br>Time spent on positive self-reflection<br><br>Ethnicity<br>Vs. White:<br>Asian	$R^2 = 0.26$<br><br>B = 2.20, 95% CI 1.45 to 2.96, p < 0.0001<br><br><br><br>B = 8.02, 95% CI 5.04 to 11.00, p < 0.0001	0.25	X^2 = 36.53, p < 0.0001

Black<br><br>Mixed<br><br>Developed new skills in physical healthcare<br><br>Developed new skills in mental healthcare<br><br>Time spent connecting with colleagues<br><br>Time spent connecting with friends and family	B = 10.64, 95% CI 7.36 to 13.92, $p < 0.0001$<br>B = 2.77, 95% CI -0.70 to 6.23, $p = 0.12$<br>B = 2.73, 95% CI 1.88 to 3.58, $p < 0.0001$<br><br>B = 0.51, 95% CI -0.25 to 1.27, $p = 0.19$<br><br>B = 1.24, 95% CI 0.37 to 2.11, $p = 0.005$<br><br>B = 1.60, 95% CI 0.73 to 2.47, $p < 0.0001$		
Model 5 Other self-care<br>Time spent on positive self-reflection<br><br>Ethnicity<br>Vs. White:<br>Asian<br><br>Black<br><br>Mixed<br><br><br>Developed new skills in physical healthcare<br><br>Developed new skills in mental healthcare<br><br>Time spent connecting with colleagues<br><br>Time spent connecting with friends and family<br><br>Time spent relaxing or doing hobbies<br><br>Time spent exercising	$R^2 = 0.27$<br><br>B = 2.21, 95% CI 1.29 to 2.96, $p < 0.0001$<br><br><br><br>B = 8.04, 95% CI 5.06 to 11.01, $p < 0.0001$<br>B = 12.06, 95% CI 8.70 to 15.43, $p < 0.0001$<br>B = 2.91, 95% CI -0.55 to 6.36, $p = 0.09$<br><br>B = 2.71, 95% CI 1.86 to 3.56, $p < 0.0001$<br><br>B = 0.53, 95% CI -0.23 to 1.29, $p = 0.17$<br><br>B = 1.29, 95% CI 0.42 to 2.16, $p = 0.004$<br><br>B = 1.59, 95% CI 0.68 to 2.50, $p = 0.001$<br><br>B = -0.24, 95% CI – 1.07 to 0.59, $p = 0.57$<br><br>B = 0.43, 95% CI -0.39 to 1.25, $p = 0.31$	0.26	$X^2 = 1.99$, $p = 0.55$
Model 6 Feeling supported	$R^2 = 0.29$	0.28	$X^2 = 27.23$, $p < 0.001$

Time spent on positive self-reflection<br><br>Ethnicity<br>Vs. White:<br>Asian<br><br>Black<br><br>Mixed<br><br>Developed new skills in physical healthcare<br><br>Developed new skills in mental healthcare<br><br>Time spent connecting with colleagues<br><br>Time spent connecting with friends and family<br><br>Time spent relaxing or doing hobbies<br><br>Time spent exercising<br><br>Felt supported by my immediate team<br><br>Felt supported by senior management<br><br>Felt supported by the UK government<br><br>Felt supported by the people of the UK	B = 2.06, 95% CI 1.24 to 2.98, $p < 0.0001$<br><br><br><br>B = 8.06, 95% CI 5.09 to 11.04, $p < 0.0001$<br>B = 12.34, 95% CI 8.97 to 15.72, $p < 0.0001$<br>B = 2.72, 95% CI -0.69 to 6.14, p = 0.12<br>B = 2.52, 95% CI 1.67 to 3.36, $p <0.0001$<br><br>B = 0.36, 95% CI -0.40 to 1.13, p = 0.35<br><br>B = 0.99, 95% CI 0.09 to 1.89, p = 0.03<br><br>B = 1.57, 95% CI 0.67 to 2.47, p = 0.001<br><br>B = -0.45, 95% CI -1.28 to 0.38, p = 0.29<br><br>B = 0.37, 95% CI -0.46 to 1.19, p = 0.38<br>B = -0.27, 95% CI -1.29 to 0.75, p = 0.60<br><br>B = 0.85, 95% CI 0.02 to 1.68, p = 0.04<br><br>B = 1.01, 95% CI 0.22 to 1.80, p = 0.01<br><br>B = 0.96, 95% CI 0.15 to 1.77, p = 0.02		
Model 7 Professional characteristics<br><br>Time spent on positive self-reflection<br><br>Ethnicity<br>Vs. White:<br>Asian	R^2 = 0.33<br><br><br>B = 1.95, 95% CI 1.10 to 2.79, $p < 0.0001$<br><br><br><br>B = 7.31, 95% CI 3.97 to 10.65, $p < 0.0001$	Adjusted R^2 = 0.30	X^2 = 50.56, $p < 0.0001$

Black	B = 11.48, 95% CI 7.93 to 15.03, p < 0.0001		
Mixed	B = 2.95, 95% CI -0.61 to 6.51, p = 0.10		
Developed new skills in physical healthcare	B = 2.15, 95% CI 1.21 to 3.08, p < 0.001		
Developed new skills in mental healthcare	B = 1.27, 95% CI 0.43 to 2.11, p = 0.003		
Time spent connecting with colleagues	B = 0.76, 95% CI -0.15 to 1.66, p = 0.101		
Time spent connecting with friends and family	B = 1.64, 95% CI 0.73 to 2.55, p < 0.0001		
Time spent relaxing or doing hobbies	B = -0.54, 95% CI -1.37 to 0.29, p = 0.199		
Time spent exercising	B = 0.59, 95% CI –0.25 to 1.42, p = 0.17		
Felt supported by my immediate team	B = -0.47, 95% CI -1.50 to 0.53, p = 0.37		
Felt supported by senior management	B = 0.99, 95% CI 0.14 to 1.84, p = 0.023		
Felt supported by the UK government	B = 0.35, 95% CI -0.46 to 1.16, p = 0.396		
Felt supported by the people of the UK	B = 1.37, 95% CI 0.55 to 2.20, p = 0.001		
Profession Vs. non-clinical			
AHPs & social workers	B = -5.43, 95% CI -8.21 to -2.65, p < 0.0001		
HCAs & support workers	B = -3.63, 95% CI -7.21 to -0.05, p = 0.047		
Medical	B = -4.49, 95% CI -8.40 to –0.58, p = 0.022		
Nursing & midwifery	B = -4.95, 95% CI -7.50 to -2.40, p < 0.0001		
Psychology & psychotherapy	B = -6.69, 95% CI -9.40 to -3.97, p < 0.0001		
Location of work Vs. Administration offices			

Community only<br><br>Inpatient (or mixed)<br><br>Healthcare sector<br>Vs. primary care:<br>Community physical health<br>Mental health<br><br>NHS Trust<br>Vs. East London<br>CNWL<br><br>Devon<br><br>NSFT	B = 0.33, 95% CI -1.35 to 2.01, p = 0.703<br>B = -0.54, 95% CI -2.94 to 1.87, p 0.661<br><br><br><br>B = -3.39, 95% CI -7.50 to 0.72, p = 0.106<br>B = -3.93, 95% CI -7.44 to -0.42, p = 0.028<br><br><br><br>B = 0.08, 95% CI -1.81 to 1.97, p = 0.934<br>B = -0.32, 95% CI -2.32 to 1.69, p = 0.755<br>B = -1.03, 95% CI -3.29 to 1.23, p = 0.370		
Model 8 Exposure to or risk of COVID-19<br><br>Time spent on positive self-reflection<br><br>Ethnicity<br>Vs. White:<br>Asian<br><br>Black<br><br>Mixed<br><br>Developed new skills in physical healthcare<br><br>Developed new skills in mental healthcare<br><br>Time spent connecting with colleagues<br><br>Time spent connecting with friends and family<br><br>Time spent relaxing or doing hobbies<br><br>Time spent exercising	$R^2 = 0.34$<br><br><br>B = 2.01, 95% CI 1.17 to 2.85, p < 0.0001<br><br><br><br>B = 6.53, 95% CI 3.18 to 9.89, p < 0.0001<br>B = 11.15, 95% CI 7.57 to 14.74, p < 0.0001<br>B = 3.17, 95% CI -0.37 to 6.72, p = 0.080<br>B = 2.03, 95% CI 1.10 to 2.96, p < 0.0001<br><br>B = 0.98, 95% CI 0.14 to 1.83, p = 0.023<br><br>B = 0.73, 95% CI -0.17 to 1.63, p = 0.112<br><br>B = 1.67, 95% CI 0.77 to 2.57, p < 0.0001<br><br>B = -0.43, 95% CI -1.25 to 0.40, p = 0.312<br><br>B = 0.78, 95% CI -0.05 to 1.62, p = 0.067	0.31	$X^2 = 15.67$, p = 0.0013<br>AIC = 5522.7<br>BIC = 5680.2

Felt supported by my immediate team<br><br>Felt supported by senior management<br><br>Felt supported by the UK government<br><br>Felt supported by the people of the UK<br><br>Profession<br>Vs. non-clinical<br><br>AHPs & social workers<br>HCAs & support workers<br>Medical<br><br>Nursing & midwifery<br><br>Psychology & psychotherapy<br><br>Location of work<br>Vs. Administration offices<br>Community only<br><br>Inpatient (or mixed)<br><br>Healthcare sector<br>Vs. primary care:<br>Community physical health<br>Mental health<br><br>NHS Trust<br>Versus East London<br>CNWL<br><br>Devon<br><br>NSFT<br><br><br>Extent of anxiety about the personal and work-	B = -0.51, 95% CI -1.54 to 0.52, p = 0.334<br><br>B = 1.07, 95% CI 0.22 to 1.91, p = 0.013<br><br>B = 0.44, 95% CI -0.36 to 1.24, p = 0.282<br><br>B = 1.32, 95% CI 0.50 to 2.15, p = 0.002<br><br><br><br><br>B = -5.41, 95% CI -8.18 to 2.63, p < 0.0001<br>B = -3.86, 95% CI -7.45 to -0.27, p = 0.035<br>B = - 4.58, 95% CI -8.49 to – 0.68, p = 0.022<br>B = -5.23, 95% CI -7.80 to -2.65, p < 0.0001<br>B = -6.32, 95% CI -9.04 to -3.60, p < 0.0001<br><br><br><br><br>B = -0.12, 95% CI -1.80 to 1.56, p = 0.890<br>B = -0.40, 95% CI -2.73 to 1.99, p = 0.741<br><br><br>B = -3.79, 95% CI -7.87 to 0.30, p = 0.069<br>B = -3.92, 95% CI -7.41 to -0.44, p = 0.027<br><br><br><br>B = 0.47, 95% CI -1.41 to 2.36, p = 0.627<br>B = -0.03, 95% CI -2.03 to 1.96, p = 0.974<br>B = -1.00, 95% CI -3.24 to 1.25, p = 0.383<br><br><br>B = 2.05, 95% CI 0.80 to 3.30, p = 0.001		

related consequences of COVID-19<br><br>Self, family or friends becoming seriously ill or hospitalised with COVID-19<br><br>Self or family CEV	 <br>B = 1.10, 95% CI -0.73 to 2.92, p = 0.239<br><br>B = 1.41, 95% CI -0.58 to 3.39, p = 0.164		
Model 9 Final model removing non-significant predictors<br><br>Time spent on positive self-reflection<br><br>Ethnicity<br>Vs. White:<br>Asian<br><br>Black<br><br>Mixed<br><br>Developed new skills in physical healthcare<br><br>Developed new skills in mental healthcare<br><br>Time spent connecting with friends and family<br><br>Felt supported by senior management<br><br>Felt supported by the people of the UK<br><br>Profession<br>Vs. non-clinical<br>AHPs & social workers<br>HCAs & support workers	$R^2 = 0.32$<br><br>B = 2.17, 95% CI 1.40 to 2.94, p < 0.0001<br><br>B = 7.03, 95% CI 3.78 to 10.27, p < 0.0001<br>B = 11.21, 95% CI 7.82 to 14.60, p < 0.0001<br>B = 2.85, 95% CI -0.65 to 6.34, p = 0.11<br><br>B = 2.13, 95% CI 1.23 to 3.04, p < 0.0001<br><br>B = 1.05, 95% CI 0.22 to 1.88, p = 0.013<br><br>B = 1.89, 95% CI 1.10 to 2.67, p < 0.0001<br><br>B = 0.89, 95% CI 0.20 to 1.58, p = 0.012<br><br>B = 1.46, 95% CI 0.70 to 2.21, p < 0.0001<br><br>B = -5.60, 95% CI -7.77 to -3.42, p < 0.0001<br>B = -3.56, 95% CI -6.69 to -0.44, p = 0.026	$R^2 = 0.31$	$X^2 = 14.05$, p = 0.297<br>AIC = 5512.7<br>BIC = 5614.7

Medical	B = - 4.92, 95% CI -8.40 to -1.44, p = 0.006		
Nursing & midwifery	B = -5.24, 95% CI -7.22 to -3.26, p < 0.0001		
Psychology & psychotherapy	B = -6.52, 95% CI -8.67 to -4.83, p < 0.0001		
Healthcare sector Vs. primary care:			
Community physical health	B = -4.16, 95% CI -8.21 to -0.11, p = 0.044		
Mental health	B = 3.68, 95% CI -7.06 to -0.31, p = 0.032		
Extent of anxiety about the personal and work-related consequences of COVID-19	B = 2.10, 95% CI 0.88 to 3.32, p = 0.001		

AHP Allied Health Professional; CEV Clinically extremely vulnerable; HCA Healthcare Assistant; NHS National Health Service; UK United Kingdom
